# Missed Capitellar Fracture Caused by Avoidance of Radiological Evaluation in Pregnancy

**DOI:** 10.1155/2018/6024057

**Published:** 2018-06-24

**Authors:** Barış Polat, Ramadan Özmanevra, Deniz Aydın, Enes Sarı, Mehmet Yalçınozan

**Affiliations:** ^1^Near East University Orthopaedics and Traumatology Department, Nicosia, Northern Cyprus, Mersin 10, Turkey; ^2^Dr. Suat Günsel Kyrenia University Orthopaedics and Traumatology Department, Kyrenia, Northern Cyprus, Mersin 10, Turkey

## Abstract

In this paper, we report a pregnant woman with a missed capitellar fracture of the elbow, who was treated successfully with open reduction and internal fixation using two headless screws. A 29-year-old 6-month pregnant woman presented to the emergency department due to a history of falling on her outstretched hand. A long-arm splint was applied without radiological evaluation due to pregnancy. She came to the orthopaedics and traumatology outpatient clinic 6 weeks after trauma and her examination after splint removal revealed pain and restriction in the elbow joint movements. Radiography was taken by using a lead shield in order to protect the fetus. Radiographs showed a displaced osteochondral capitellar fracture. Using the posterolateral approach as described by Kocher, the fracture was fixed using headless canulated compression screws. The follow-up examination showed excellent functional and radiological results. Radiological evaluation should not be avoided in case of obvious fracture findings after trauma even in case of pregnancy. It is also highlighted that good results in terms of union and functional recovery can be achieved with open reduction and headless compression screw fixation followed by early rehabilitation even in delayed treatment of capitellum fractures.

## 1. Introduction

Isolated fractures of the humeral capitellum are rare and commonly missed in the initial examination [1]. Patients with capitellar fractures mostly present with painful swelling of the elbow. Physical examination findings involve painful restriction of joint movements and locking. Treatment options for capitellum fracture consist of closed reduction and long-arm splint, fragment excision, open reduction and internal fixation, and arthroscopic fixation. Fixation can be achieved with Kirschner wires (K-wires), cannulated cancellous screws, or headless compression screws. Despite this diversity of treatment, headless compression screws via open reduction and internal fixation provide excellent compression at the fracture site and stable fixation with the least damage to the articular surfaces. Furthermore, early mobilization can be initiated and it is not necessary to remove the hardware later [2].

Fracture management in pregnant patients is challenging. It is a common belief that plain radiographs may cause high levels of radiation exposure both for the pregnant patient and fetus. To the author's knowledge, no cases of missed capitellar fracture due to pregnancy have been reported in the English literature until now. We report a pregnant patient with a missed capitellar fracture of the elbow, who was treated successfully with open reduction and internal fixation using two headless screws.

## 2. Case Presentation

**Figure 1:**
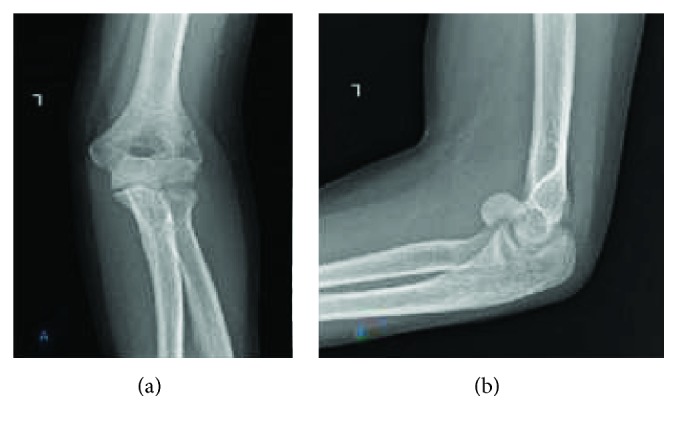
Preoperative left-elbow plain X-rays. (a) Anteroposterior and (b) lateral.

**Figure 2:**
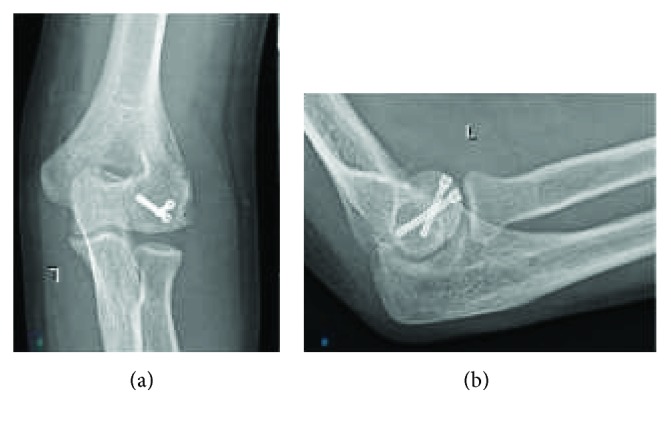
Postoperative control X-rays (third month). (a) Anteroposterior and (b) lateral.

**Figure 3:**
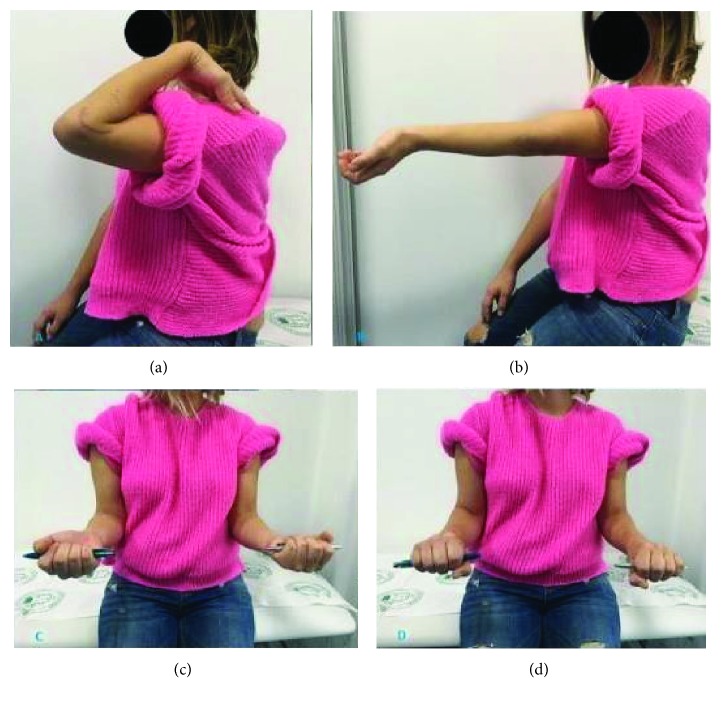
Postoperative elbow functional assessment (sixth month). (a) Flexion, (b) extension, (c) supination, and (d) pronation.

A 29-year-old, right-handed, 6-month pregnant woman presented to another hospital's emergency service with a history of falling on her outstretched left hand. X-ray was not administered to her due to pregnancy. She was treated with a long-arm splint. After 6 weeks, she was admitted to our outpatient clinic with pain and swelling around the elbow. Her elbow flexion-extension range was measured as 60/100 and supination-pronation was measured as 45/45. The elbow varus-valgus stress tests were normal. There was no neurovascular deficit. Plain X-rays were taken by using a lead shield to protect the fetus. Radiographs showed a Bryan and Morrey type I osteochondral capitellar fracture that displaced anterosuperiorly (Figure 1). There was no concomitant injury in the forearm, radius, or distal radioulnar joint. A computerized tomography (CT) scan was not performed due to her pregnancy. Open reduction and internal fixation was planned for the patient. The patient was consulted with an obstetrician preoperatively. An informed patient's consent was obtained for emergency caesarean delivery in case of acute decompensation of the fetus during surgery. The fetal heart was monitored during surgery and operation was completed without any complications. RIVA (regional intravenous anaesthesia) was applied. The patient was operated in a supine position under tourniquet control. Using the posterolateral approach as described by Kocher, the fracture was fixed. Headless cannulated compression screws (3.0 mm Barouk screws, DePuy, Lyon, France) were used for fixation. A flouroscopy was taken at the end of the surgery, using a lead shield to protect the uterus. The elbow was immobilized using a posterior long-arm splint for 3 days to prevent swelling. This was followed by a progressive elbow mobilization program guided by a physiotherapist. She was called for clinic control at 1, 2, 3, 6, and 12 months. She gave birth to a healthy baby three months after the operation. At this time, the fracture site was considered to be healed based on radiographic appearance of the fracture and absence of any pain on movement during the clinical evaluation (Figure 2). She attained 135 degrees of flexion and full extension (0–135). Supination-pronation was measured at 180 degrees three months after surgery with no further complications. Her clinical examination revealed a perfect result with a full pain-free range of motion at the 6-month (Figure 3) and 12-month follow-ups. Informed consent was obtained from the patient for the publication of this report, related pictures, and radiographies.

## 3. Discussion

Patients with capitellar fractures often present with findings such as swelling, severe painful limitation of movement, and joint locking. Every patient who is admitted to hospital with these examination findings require radiological evaluation, even if the patient is pregnant. Capitellum fractures can easily be misdiagnosed in an elbow anteroposterior X-ray due to distal humerus and capitellum overlap. Lateral elbow X-rays are more effective for determining fractures [1, 2]. CT scans are recommended to define the medial extent of the fracture, articular impaction, and metaphyseal or condylar comminution [3]. CT maybe helpful for fracture classification and preoperative planning for the choice of internal fixation implants. Despite the benefits that CT offers, it is not preferred in pregnant patients.

An untreated displaced capitellar fragment undergoes changes resulting from bony resorption to the bony proliferation and obliterates the radial fossa [2]. This condition triggers degenerative arthritis and heterotropic ossifications. Furthermore, the nonanatomic position of the displaced bone fragment may cause elbow impingement, stiffness, or decreased elbow range of motion. A variety of methods have been described to treat capitellum fractures. These include closed reduction, excision, open reduction with internal fixation, arthroscopic reduction with fixation, and prosthetic replacement [2, 4–7]. Closed reduction and immobilization have been reported in several previous studies. The long period of immobilization, increased malunion and nonunion rates, and restricted elbow range of motion are disadvantages of this treatment [6]. Capitellar fragment excision is also a simple and old treatment method. In a systematic review, 23 patients underwent excision of their capitellar fragments. After a mean of 25 months of clinical follow-up, the patients lost an average of 26 degrees of range of ulnohumeral joint motion compared with the contralateral side. Furthermore, 47% of the patients reported minimal to moderate elbow pain [4]. Open reduction and internal fixation with cannulated screws is the most commonly used and the most appropriate treatment method. Headless cannulated compression screws can be placed in an anteroposterior or posteroanterior direction. The proximal edge of the screw should be embedded in the subchondral bone in order to prevent severe cartilage damage and symptomatic intra-articular implant. Additionally, it does not require subsequent removal [2, 4, 5].

In this present report, the importance of the early progressive elbow mobilization program guided by the physiotherapist is also emphasized. In a previous study, elbow immobilization for a three-week period and rehabilitation of the joint was unable to restore the complete range of motion, even if a stable open reduction internal fixation was achieved [8]. Most authors suggest the use of a hinged brace during the first month after the surgery, although the authors of this paper believe that fixation provided by two compression screws is adequate for early mobilization without a hinged brace.

An intra-articular capitellar fragment may maintain its viability with diffusion from the synovial fluid. Early fixation is important for the success of the treatment. Traditionally, in delayed diagnoses or old fractures, excision of the fragment is recommended because of the risk of osteonecrosis [7]. In one study, patients were treated on the day of presentation or at an average of 5.7 months after injury by excision. The average range of motion in the ulnohumeral joint were 141 degrees in the early group and 91 degrees in the late group. All patients treated with excision suffered some residual pain at the final follow-up [7]. Because of these unsatisfactory results of treatment with excision, three patients with capitellar fracture were treated with open reduction and internal fixation with augmentation using a bone graft. All patients showed good union and functional results [5]. Satisfactory results can be obtained with open reduction and internal fixation with or without using a bone graft even if the capitellar fracture is neglected or treatment is delayed.

Fracture management in pregnant patients can be challenging. In utero exposure to ionizing radiation can result in detrimental consequences to the fetus. These effects are related to the dose applied and the gestational age of the fetus. The use of radiographs and CT are the main potential sources of ionizing radiation in the evaluation and management of orthopaedic injuries. Significant care must be taken to protect the fetus from exposure. In case of high clinical suspicion of fracture in a traumatized pregnant patient, ultrasound (USG) may be used for radiological evaluation. USG is a noninvasive, fast, and cheap radiological method to evaluate traumatized patients. Considering the potential disadvantages of USG (radiologist dependent, low sensitivity for bony lesions), the X-ray is still superior in the diagnosis of a fracture.

In pregnant patients, the primary goal in fracture fixation should be to use the fixation technique that requires the least amount of radiation without compromising fracture care. When the benefits and risks of different surgical techniques are evaluated, clinician experience plays an important role.

Contrary to popular belief, plain radiography exposes the pregnant woman and fetus to minimal radiation [9]. However, unnecessary irradiation should be avoided, and the lead shielding should be used to protect the fetus. If the patient is not lead-shielded, the fetus may receive 30% of the dose administered to the mother [9]. Fluoroscopy use must be minimized, but it should not be avoided if it is necessary for fracture reduction and fixation.

There is no conclusive evidence that the type of anaesthesia, whether it be regional or general, is safer than the other. Elective surgical procedures in the pregnant patient should be delayed until after delivery. When general anaesthesia is necessary, ACOG recommends intraoperative fetal monitoring of all viable fetuses whenever possible. Additional recommendations include having an obstetrician available and obtaining patient consent for emergency caesarean delivery before nonobstetric surgery in case of emergency [10]. In this case, the anaesthetist was consulted and the decision was made to use RIVA anaesthesia. The anaesthetist performed fetal heart monitoring during the surgery and the obstetrician was consulted before and after the surgery.

Delayed diagnosis of a humerus capitellum fracture in a pregnant patient was reported in this case. Radiological evaluation should not be avoided in case of obvious fracture findings after trauma even in case of pregnancy. It is also highlighted that good results in terms of union and functional recovery can be achieved with open reduction and headless compression screw fixation followed by early rehabilitation even in delayed treatment of capitellum fractures.
